# Supplementary material for: Exploratory Study to Evaluate the Impact of Interim PET/CT Assessment in First-Line Follicular Lymphoma
Source: Cancers (Basel). 2025 Mar 21;17(7):1065. doi: 10.3390/cancers17071065 (PMC11988115; doi:10.3390/cancers17071065)
Supplement: Supplementary file 1 [file cancers-17-01065-s001.zip › Supplementary figures.pdf]

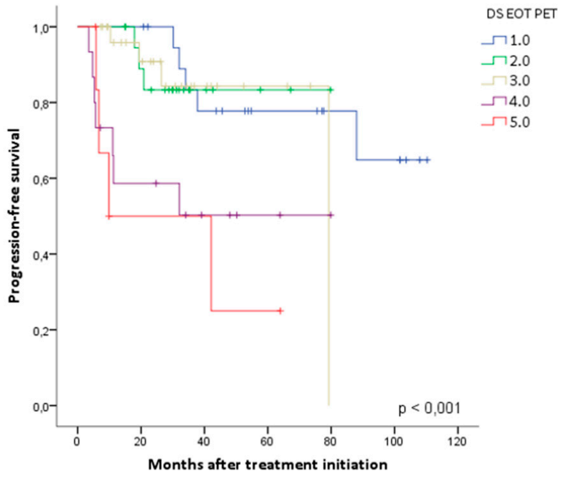

Supplementary Figure S1. Progression-free survival rates according to Deauville score at end-of-treatment PET.

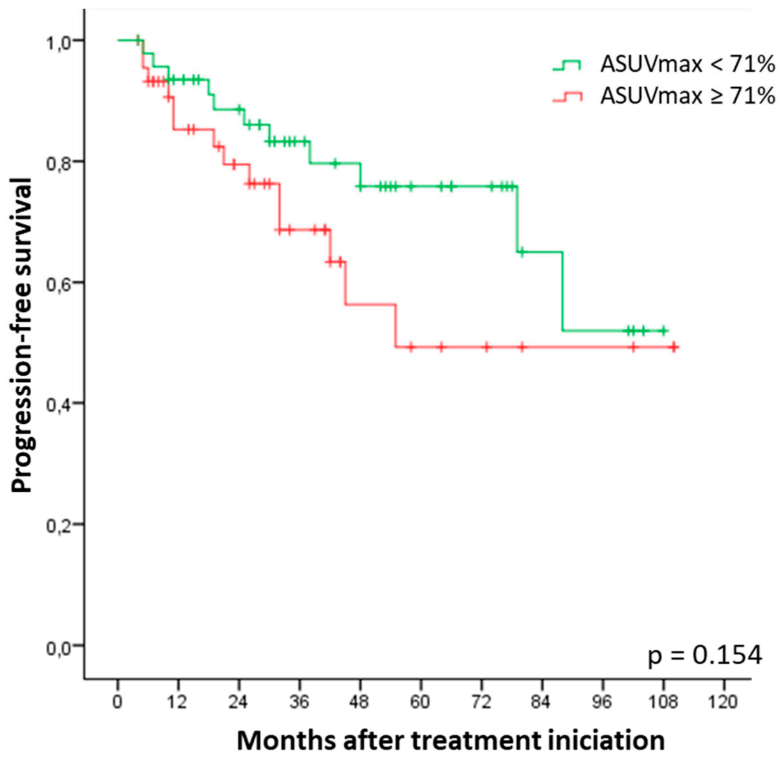

Supplementary Figure S2. Progression-free survival rate according to ΔSUVmax between basal and interim PET.

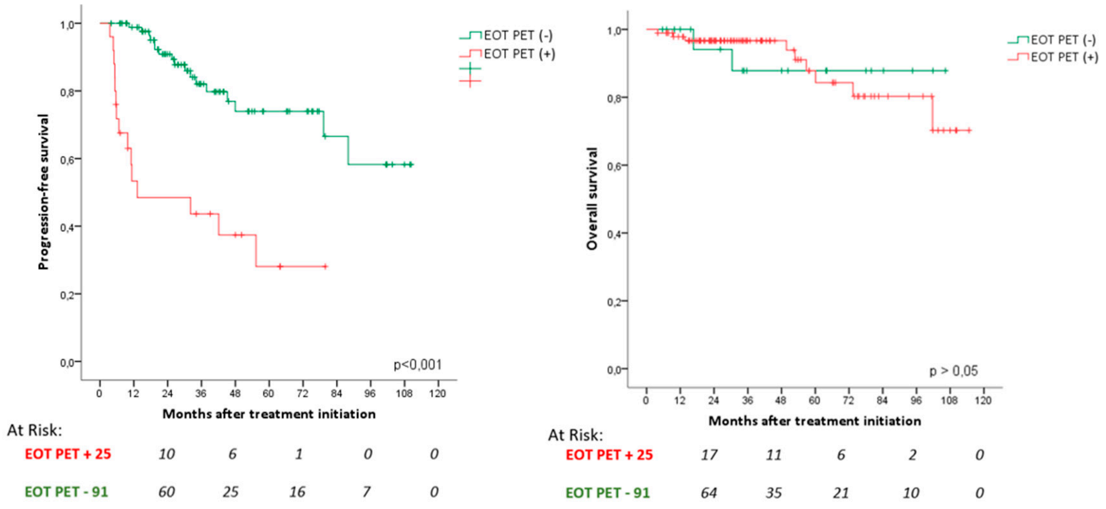

(A) (B)

Supplementary Figure S3. (A) Progression-free survival rate according to EOT PET review (threshold ≥ 4). **(B)** Overall survival rate according to EOT PET review (threshold ≥ 4).
